# Smartphone-assisted guided self-help cognitive behavioral therapy for young people with distressing voices (SmartVoices): study protocol for a randomized controlled trial

**DOI:** 10.1186/s13063-022-06846-0

**Published:** 2022-10-23

**Authors:** Marialuisa Cavelti, Janko M. Kaeser, Stefan Lerch, Stephanie Bauer, Markus Moessner, Thomas Berger, Mark Hayward, Michael Kaess

**Affiliations:** 1 University Hospital for Child and Adolescent Psychiatry and Psychotherapy, University of Bern, Bern, Switzerland; 2 Centre for Psychotherapy Research, University Hospital Heidelberg, Heidelberg, Germany; 3 Department of Clinical Psychology and Psychotherapy, University of Bern, Bern, Switzerland; 4 Research & Development Department, Sussex Partnership NHS Foundation Trust, Hove, UK; 5 Department of Psychology, University of Sussex, Brighton, UK; 6 Department of Child and Adolescent Psychiatry, Center for Psychosocial Medicine, University of Heidelberg, Heidelberg, Germany

**Keywords:** Auditory hallucinations, Voices, Therapy, Treatment, Ecological momentary assessment, Ecological momentary intervention, Experience sampling method, Just-in-time adaptive interventions, Adolescents, Youth

## Abstract

**Background:**

The long-standing view that auditory verbal hallucinations (AVH) or hearing voices is a sign of schizophrenia has been challenged by research demonstrating that they lie on a continuum ranging from normal to pathological experience related to distress and need for care. Hearing voices is more prevalent in adolescence than in later life, and hearing voices during adolescence indicates a risk for severe psychopathology, functional impairments, and suicide later in life. While there is increasing evidence for the efficacy of cognitive behavioral therapy for voices (CBTv) in adults with schizophrenia, research on psychological treatments for youth with distressing voices has been scarce. The aim of the current study is to examine the efficacy of CBTv, delivered using smartphone-based Ecological Momentary Assessment Intervention (EMI) in a transdiagnostic sample of youth.

**Methods:**

This is a superiority randomized controlled trial comparing 8 weeks of CBTv-based EMI in addition to treatment as usual (TAU) versus TAU only. TAU covers both no treatment and any form of psychiatric/psychological treatment. In the EMI condition, participants will be prompted twice a day to complete an EMA survey, and receive one intervention proposal per assessment. One-hundred fifty-four youth aged 14–25 years with distressing voices will be recruited from psychiatric clinics, local private practices, internet forums, and advertisements in print and social media. Before and after the intervention phase, participants will undergo a 9-day EMA. Single-blinded assessments will be conducted at baseline (T0) and at 3-month (T1) and 6-month (T2) follow-up. The primary outcome is the distress dimension of the Auditory Hallucinations subscale of the Psychotic Symptom Rating Scales at T1. Secondary outcomes include perceived hostile intention, power, and dominance of voices, passive, aggressive, and assertive relating to voices, and negative core beliefs about the self.

**Discussion:**

Adolescence provides a crucial window of opportunity for early intervention for hearing voices. However, youth are notoriously reluctant help-seekers. This study offers a low-intensity psychological intervention for youth with distressing voices beyond diagnostic boundaries that, using a mobile technology approach, may match the treatment preferences of the generation of “digital natives.”

**Trial registration:**

German Clinical Trials Register DRKS00026243. Registered on 2 September 2021

**Supplementary Information:**

The online version contains supplementary material available at 10.1186/s13063-022-06846-0.

## Administrative information

The numbers in curly brackets in this protocol refer to SPIRIT checklist item numbers. The order of the items has been modified to group similar items (see http://www.equator-network.org/reporting-guidelines/spirit-2013-statement-defining-standard-protocol-items-for-clinical-trials/).

**Table:** 

Title {1}	Smartphone-assisted guided self-help cognitive behavioral therapy for young people with distressing voices (SmartVoices): Study protocol for a randomized controlled trial
Trial registration {2a and 2b}.	German Clinical Trials Register (DRKS00026243), Registered on 2 September 2021
Protocol version {3}	October 19, 2022; protocol version 1.2
Funding {4}	The study is funded by a grant from the Swiss National Science Foundation (SNSF; PZOOP1_193279 / 1) and a grant from the Ebnet-Foundation in Switzerland.
Author details {5a}	^1^ University Hospital for Child and Adolescent Psychiatry and Psychotherapy, University of Bern, Bern, Switzerland ^2^ Centre for Psychotherapy Research, University Hospital Heidelberg, Heidelberg, Germany ^3^ Department of Clinical Psychology and Psychotherapy, University of Bern, Bern, Switzerland ^4^ Research & Development Department, Sussex Partnership NHS Foundation Trust, Hove, United Kingdom ^5^ Department of Child and Adolescent Psychiatry, Center for Psychosocial Medicine, University of Heidelberg, Heidelberg, Germany ^6^ Department of Psychology, University of Sussex, Brighton, United Kingdom
Name and contact information for the trial sponsor {5b}	Marialuisa Cavelti, University Hospital for Child and Adolescent Psychiatry and Psychotherapy, Bolligenstrasse 111, 3000 Bern 60, Switzerland
Role of sponsor {5c}	The sponsor designed the study and is responsible for the collection, management, analysis, and interpretation of data, writing of the report, and the decision to submit the report for publication.

## Introduction

### Background and rationale {6a}

Auditory verbal hallucinations (AVH)—or hearing voices—are real-seeming auditory experiences in the form of spoken language that occur without a corresponding sensory input [1]. While hearing voices has long been considered a sign of a psychotic disorder such as schizophrenia, recent evidence suggests that they lie on a continuum ranging from normal to pathological experience related to distress and need for care [2]. Auditory hallucinations are more prevalent in adolescence (12.4%) than in later life (5.8% in middle and 4.5% in late adulthood) [3]. Although the majority of hallucinatory experiences in adolescence is transient [4], persistence can cause high levels of emotional distress and represent a risk marker for a wide range of negative outcomes in the long term (i.e., psychotic and non-psychotic mental disorders, multimorbidity, impairments in psychosocial functioning, and suicide [5–10]). Therefore, adolescence provides a crucial window of opportunity for early intervention for hearing voices in order to prevent suffering and disability in the affected young people and improve their life trajectories.

Cognitive behavioral therapy for psychosis (CBTp) is recommended for the treatment of individuals with psychotic disorders such as schizophrenia. In contrast to CBTp that targets positive symptoms (i.e., delusions and hallucinations) more broadly, CBT for voices (CBTv) is a symptom-specific development that focuses on key processes assumed to contribute to the emergence and persistence of distress and disability associated with voice hearing. Based on the continuum hypothesis of hearing voices [2], CBTv intervenes at the level of the individual “meaning-making” by targeting appraisals of voices [11] that are typically perceived as powerful, dominant, and intrusive [12, 13]. In more recent times, self-related and interpersonal variables have been included as additional or alternative treatment targets in CBTv approaches, as evidence indicates that people who are distressed by their voices typically feel powerless and inferior to their voices and respond relationally from a position of subordination (e.g., passively or aggressively) [12, 13]. Interestingly, relational experiences with the voices seem to mirror relational experiences within the wider social context, such that similar styles of relating are evident within the relationships with voices and other people [14–16]. A recent systematic review of eight randomized controlled trials (RCT) addressing hearing voices supports the general efficacy of CBTv in reducing voice-related distress or problematic behavior (e.g., compliance with voices), with effect sizes ranging from small (Cohen’s *d* = 0.44) to very large (*d* = 1.78). The change of the power imbalance between the voice and the voice-hearer was a common component of all included studies and may, thus, represent a key mechanism of change for all CBTv approaches [17].

These data suggest that CBTv could be a promising option for early intervention for young people with distressing voices. However, there are several challenges: First, the accessibility of CBTv is limited, due to scarcity of resources, geographic barriers, and ambivalent attitudes of patients and clinicians [18]. Second, although CBTv is potentially applicable to hearing voices beyond diagnostic boundaries [11, 18], its efficacy for voice-hearers with non-psychotic disorders or without any psychiatric diagnosis has rarely been investigated [17]. Third, as CBTv was developed for adults, little is known whether or not it is suitable for the treatment of young people with voices who might have specific developmental needs and distinct treatment preferences (e.g., flexible access, non-stigmatizing support, preference for self-reliance) [19, 20]. Fourth, young people with mental health issues show low use of face-to-face treatments [19, 21–23]. This has led to the request for a transformation of traditional mental health services to better match the needs and preferences of the youth [20]. The usage of digital technologies for treatment delivery may be part of the solution.

### Objectives {7}

The aim of the current study is to examine the efficacy of CBTv, delivered using smartphone-based Ecological Momentary Interventions (EMI) in a transdiagnostic sample of youth with distressing voices. The usage of smartphone-based EMI, also known as just-in-time adaptive interventions [24], provides several advantages: First, built upon Ecological Momentary Assessment (EMA; also called experience sampling method), the interventions can be individually tailored to the person’s momentary experiences and symptoms. Second, the interventions occur in the context of daily life, therefore bridging the gap between the clinical setting and the real world. Third, the usage of mobile technology for treatment delivery provides an opportunity for low-intensity and time- and place-unlimited support. This may be particularly relevant for young people who prefer short and uncomplicated interventions and find smartphones an appealing medium to deliver mental health treatments [25, 26]. In addition, mobile interventions can present a promising option where specialized face-to-face treatments are not available. Finally, EMI facilitate the examination of whether changes in putative psychological mechanisms ultimately result in changes in the symptoms they are supposed to be causing (i.e., interventionist causal model approach), therefore contributing to a better understanding of the mechanisms of change underlying CBTv [27]. A recent systematic review on EMI for people with psychotic disorders reported generally positive acceptability and feasibility ratings and provided promising evidence of improved clinical outcomes [28]. A first symptom-specific EMI trial for adults with persistent and distressing voices demonstrated its potential for improving comping with voices [29].

The main hypotheses of the current study are as follows: First, an 8-week treatment as usual (TAU) + CBTv-based EMI is more effective in reducing voice-related distress compared with TAU only. Second, the improvement attributable to the CBTv-based EMI will be maintained during the 6-month follow-up.

### Trial design {8}

**Fig. 1:**
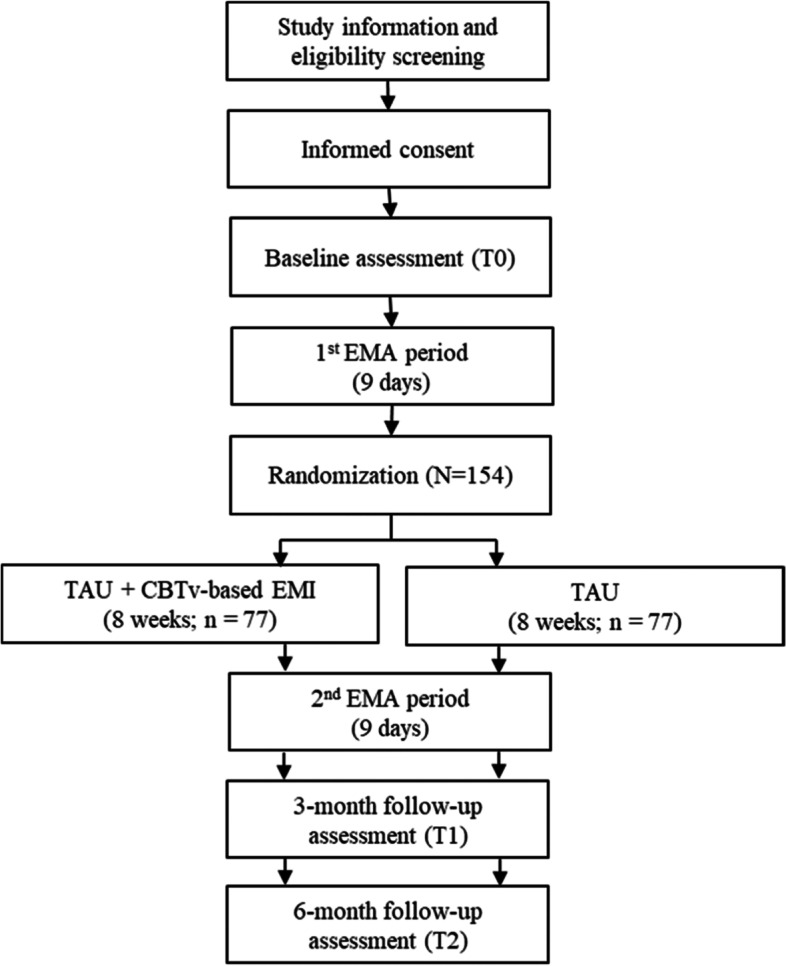
Illustration of the participant flow through the study. EMI, Ecological Momentary Intervention; CBTv, cognitive behavioral therapy for voices; TAU, treatment as usual

This is a prospective, multicenter, assessor-blinded, two-armed, parallel-group, superiority RCT, using a 1:1 allocation. After providing written informed consent, participants will undergo a baseline assessment (T0) followed by a 9-day EMA. Afterwards, participants will be randomized to the intervention group or the control group. For the next 8 weeks, the intervention group will receive a CBTv-based EMI in addition to TAU, while the control group will receive TAU only. Afterwards, both groups will undergo another 9-day EMA, followed by a 3-month follow-up assessment (T1), and a 6-month follow-up assessment (T2). Figure 1 displays the Consolidated Standards of Reporting Trials (CONSORT) flow diagram of the study procedure.

## Methods: participants, interventions, and outcomes

### Study setting {9}

Participants will be recruited from the following psychiatric clinics, located in the Canton of Bern, Switzerland: (1) Universitätsklinik für Kinder- und Jugendpsychiatrie und Psychotherapie, Universitäre Psychiatrische Dienste Bern (University Hospital for Child and Adolescent Psychiatry and Psychotherapy, University Psychiatric Services Bern); (2) Universitätsklinik für Psychiatrie und Psychothearpie, Universitäre Psychiatrische Dienste Bern (University Hospital for Psychiatry and Psychotherapy, University Psychiatric Services Bern); (3) Privatklinik Wyss in Münchenbuchsee (Private Psychiatric Clinic Wyss); (4) Privatklinik Meiringen (Private Psychiatric Clinic Meiringen); and (5) Psychiatriezentrum Münsingen (Psychiatry Center Münsingen). Additionally, participants will be recruited from local psychiatric or psychological private practices as well as via the webpage www.online-therapy.ch from the University of Bern, relevant internet forums, and advertisements in print and social media.

### Eligibility criteria {10}

Participants meeting the following criteria will be included in the trial: (1) aged 14 to 25 years; (2) experiencing current and persisting voices defined as a score of 1 (“voices occur at least once a week”) or above on the frequency item (no. 1) of the Psychotic Symptom Rating Scales-Auditory Hallucinations subscale (PSYRATS-AH) [30]; (3) experiencing distress due to voices, defined as a score of 2 (“a minority of voices is distressing (< 50%)”/“voices are moderately distressing”) or above on either of the distress items—amount of distress (no. 8) or intensity of distress (no. 9)—of the PSYRATS-AH; and (4) written informed consent. Exclusion criteria are as follows: (1) insufficient conduct of German; (2) hearing voices with an organic cause (e.g., brain disease or injury); (3) acute risk of harm to self or others that requires immediate crisis intervention; (4) inability to give informed consent and/or adhere with the study procedure; (5) currently receiving face-to-face psychological therapy that specifically addresses hearing voices (e.g., CBTp); and (6) intellectual disability.

### Who will take informed consent? {26a}

Potential participants will be contacted by a member of the research team to provide a participant information sheet with sufficient information to make an informed decision, discuss the study, screen for eligibility, and obtain written informed consent. The meeting can take place in person or by phone. If the latter is the case, the participant information sheet and informed consent form will be sent by email. The researchers will make sure that potential participants have enough time to consider whether or not they want to participate in the study. According to Swiss law, adolescents from the age of 14 are allowed to sign the informed consent form themselves (no parental consent needed). For participants aged 14–17 years, parents or legal guardians will receive a separate participant information sheet.

### Additional consent provisions for collection and use of participant data and biological specimens {26b}

Not applicable—this trial does not involve collecting biological specimens.

### Interventions

#### Explanation for the choice of comparators {6b}

In this two-armed, parallel group, superiority RCT, TAU was chosen as the control group in order to examine whether CBTv-based EMI added to standard treatment is superior to standard treatment alone in reducing voice-related distress. Thereby, TAU was defined as including both no psychological/psychiatric treatment at all and any form of psychological/psychiatric treatment. While this strategy will result in a heterogeneous TAU group requiring detailed description, it has the advantage that youth who are not in treatment yet and for whom the smartphone-based intervention represents an attractive, low-intensity option can also be included in the study.

#### Intervention description {11a}

##### CBTv-based EMI

Participants allocated to the intervention group will receive 8 weeks of EMI based on CBTv in addition to TAU. The EMI will have a time-contingent design with a semi-random sampling schedule. Participants will be prompted twice a day to complete an EMA survey on their smartphone. Prompts will occur randomly within two time intervals occurring between 8:00am and 12:00pm and between 2:30pm and 6:30pm, respectively. Each EMA survey will take approx. 2–3 min to complete and assesses the occurrence of voices since the previous assessment; perceived distress of voices; beliefs about voices; passive, aggressive, and assertive relating to voices; whether they had social contact since the last assessment; passive, aggressive, and assertive relating to other people; self-esteem; whether they have used the therapeutic strategies proposed at the previous assessment; and perceived helpfulness of the proposed therapeutic strategies or reasons of non-use, respectively. A full list of the items is provided in Additional file 1. Based on the individual responses to the EMA survey, participants will receive one intervention proposal per assessment to try out until the next assessment.

The EMA and EMI are implemented using the web-based software program ASMO (Ger.; Assessment und Monitoring psychischer Gesundheit; asmo.online) developed by the Center for Psychotherapy Research, University Hospital Heidelberg, Germany [31]. The software has been used in numerous collaborative projects (e.g., [32, 33]) and will be adapted to meet the requirements of the current trial. As soon as participants are registered on the program, they are automatically notified using text messaging via the Short Message Service (SMS) whenever an EMA is due. They can click on the link in the SMS and are automatically directed to the internet platform where they complete the EMA survey. A software program automatically analyzes the incoming messages based on a pre-defined algorithm (e.g., with respect to the occurrence of voices since the last assessment or the participant’s momentary (functional versus dysfunctional) beliefs about voices). Based on this evaluation, the program automatically selects a feedback message from a pool of pre-formulated statements and the message is presented to the participant in response to their report. In addition, participants can access the internet platform to receive therapeutic information and exercises at any time.

Before the start of the 8-week EMI, a single, individual introduction session will take place. In this session, participants will get familiarized with the rationale of the CBTv and receive a technical introduction to ensure they understand how to use the EMI. They will be encouraged to contact the research team whenever they experience difficulties during the intervention phase. After the 8-week EMI, a single, individual closing session will take place in order to explore what participants have learnt from the intervention and what they will take away for the future.

The intervention and the introduction and closing sessions are informed by the “Overcoming Distressing Voices” self-help book by Mark Hayward [34]. Specifically, Chapter 1 (“Understanding voices”), Chapter 2 (“Self-esteem and hearing voices”), Chapter 3 (“Relationships with voices and other people”), Chapter 4 (“Coping with voices”), Chapter 5 (“Changing beliefs about voices”), Chapter 6 (“Overcoming low self-esteem and distressing voices”), Chapter 7 (“Changing our relationship with voices and other people”), and Chapter 8 (“Moving forwards”) are translated to German and adapted for the use in the current study.

##### Treatment as usual (TAU)

TAU can include both no psychological/psychiatric treatment at all and any form of psychological/psychiatric treatment. No restrictions are imposed on the setting (i.e., inpatient vs. outpatient treatment) or type of treatment (i.e., psychological or pharmacological). Treatment may include the following elements: psychiatric case management, psychotropic medication, supportive counseling sessions, psychosocial interventions (e.g., social work, peer support), and individual, family, or group psychological therapies. Detailed information on the type and extent of the TAU at baseline (T0) as well as on changes in TAU over the study period (T1, T2) will be collected.

#### Criteria for discontinuing or modifying allocated interventions {11b}

Participants can be excluded from the study if the data of the baseline EMA reveals that voices primarily occur under the influence of alcohol and/or drug use or if further participation would pose a risk to them.

#### Strategies to improve adherence to interventions {11c}

Participants adherence with the assessment schedules of the two EMA periods and the EMI will be monitored electronically by the research team, using the administration tool of the web-based software program ASMO. Participants showing a significant drop in their response rate will be contacted to clarify the reasons of non-adherence, boost motivation, and offer problem-solving and support when needed.

A high standardization of the introduction and closing sessions will be facilitated by the usage of a detailed session manual and intensive training and supervision of the researchers conducting the session.

#### Relevant concomitant care permitted or prohibited during the trial {11d}

Individuals who already receive psychological therapy that specifically targets hearing voices (e.g., face-to-face CBTp) cannot participate in the study. No other restrictions are imposed on the TAU.

#### Provisions for post-trial care {30}

Participants who are not yet receiving standard treatment and who express an interest in it at the end of the study will receive information about suitable mental health services in the area where they live.

In the unlikely event of study-related damages or injuries, the liability of the Universitären Psychiatrischen Dienste (University Psychiatric Services) Bern, Switzerland, will provide compensation, except for claims that arise from misconduct or gross negligence.

### Outcomes {12}

The primary outcome of the study is the change in the Distress dimension of the Psychotic Symptom Rating Scales Auditory Hallucinations subscale (PSYRATS-AH) [30] between the baseline (T0) and the 3-month follow-up (T1) assessments. Reducing the level of psychological distress due to voice-hearing (instead of reducing hearing voices per se) is the main therapy aim of CBTv and the PSYRATS-AH has been widely used in clinical trials investigating the efficacy of CBTv in the past [17, 35, 36]. Additionally, distress is the prioritized outcome by patients with voices and is related to dysfunction [37].

Secondary outcomes of the study include the change in (1) voice-related distress (distress dimension of the PSYHRATS-AH) between T0 or T1 and the 6-month follow-up (T2); (2) perceived hostile intent and power of voices (Persecutory Beliefs subscale of the Revised Beliefs About Voices Questionnaire; BAVQ-R) [38]; and (3) perceived voice dominance and intrusiveness (Voice and You-Revised; VAY-R) [39], passive, aggressive, and assertive relating to voices (Approve questionnaires) [40], and negative core beliefs about the self (Brief Core Schema Scales, BCSS) [41] between T0, T1, and T2. Appraisals of and relating styles to voices as well as core beliefs about the self were chosen as secondary outcomes because they represent potential mediators of the efficacy of the CBTv-based EMI [35, 42].

Other outcomes include changes in (1) severity of depression, anxiety, and stress (Depression, Anxiety and Stress Scale-21 (DASS-21) [43]); (2) overall illness severity (Clinical Global Impression Scale-Severity (CGIs) [44]); (3) psychosocial functioning (Social and Occupational Functioning Assessment Scale (SOFAS) [45]); and (4) quality of life (Quality of Life Enjoyment and Satisfaction Questionnaire (Q-LES-Q-18) [46]) between T0, T1, and T2.

### Participant timeline {13}

The participant timeline is depicted in Fig. 1 and will be reported in line with the Consolidated Standards of Reporting Trials 2013 statement [47].

### Sample size {14}

Sample size calculation was based on the expected difference between the TAU + CBTv-based EMI group and the TAU group in the severity of the PSYRATS-AH Distress dimension between the baseline (T0) and the 3-month follow-up (T1) assessments. Pilot studies examining the efficacy of the CBTv that is adopted for use as EMI in the current trial reported large effect sizes (Cohen’s *d* ranging between 1.3 and 1.78) [35, 42]. Given that these studies involved face-to-face therapy and that effect sizes are likely to be reduced in a full-scale trial, a medium effect size is targeted in the current study. A power-analysis for an analysis of variance with two groups (TAU + CBTv-based EMI, TAU), and two repeated measures (T0, T1) and within-between interaction (group x time), was calculated using G*Power 3.1.9.4 [48]. Assuming a medium effect size of *f* = 0.25, a power of 0.80, and an alpha level of .05, a sample size of *N* = 128 is required. Including an expected attrition rate of 20%, the required total sample size for the RCT is *N* = 154, with 77 participants in each treatment arm.

### Recruitment {15}

Participants will be recruited through referrals from clinical practitioners from the host clinics and clinical psychologists and psychiatrists in private practice as well as via the webpage www.online-therapy.ch from the University of Bern, internet forums, and advertisements in print and social media.

## Assignment of interventions: allocation

### Sequence generation {16a}

Randomization will be performed by the biostatistician of the University Hospital for Child and Adolescent Psychiatry and Psychotherapy, University of Bern, Switzerland (SL). Minimization, a covariate adaptive randomization, will be applied, which sequentially assigns each new participant to one of the two treatment arms by taking into account previous assignments of participants and specific covariates. This method ensures balance in important prognostic factors without the pitfalls of stratified randomization [49]. Imbalance is calculated according to Pocock and Simon [50]. In this trial, minimization will balance whether (a) voices are experienced daily or not (PSYRATS-AH item 1 (frequency) score 1 versus ≥2) and (b) participants receive any psychiatric/psychological treatment or not, because these variables are predicted to have an effect on treatment response and should therefore be balanced across groups.

### Concealment mechanism {16b}

The baseline data of each new participant including the covariates that will be used for randomization will be entered into REDCap (Research Electronic Data Capture) hosted at the University Bern, Switzerland [51, 52] (see below for more information on data management using REDCap). The data will then be exported by the biostatistician of the University Hospital for Child and Adolescent Psychiatry and Psychotherapy, University of Bern, Switzerland (SL), to STATA 17 [53]. He will allocate new participants to TAU + CBTv-based EMI or TAU based on the algorithm described by Pocock and Simon [50]. The treatment assignment will be entered by the biostatistician into REDCap in a manner that is hidden from the blinded assessors.

### Implementation {16c}

The principal investigator (MC) will be notified by the biostatistician (SL) about a participant’s group allocation. The principal investigator will then inform the member of the research team (JK) who is responsible for forwarding the information to the participant and conduct the introduction and closing sessions for those allocated to the TAU + CBTv-based EMI group.

## Assignment of interventions: blinding

### Who will be blinded {17a}

Members of the research team who will conduct the 3-month (T1) and the 6-month follow-up (T2) assessments will be blinded. Success of blinding will be assessed by asking researchers conducting T1 and T2 assessments to guess which condition the participant was allocated to and indicate their confidence. It is not feasible to blind participants to their allocation due to the design of the trial. Participants will be asked not to reveal their group allocation to the researcher conducting the T1 and T2 assessments. Additionally, researchers conducting the T1 and T2 assessments will be shielded from discussion of participants. Accidental unblinding of researchers conducting the T1 and T2 assessments will be recorded and addressed by repeating the assessment as soon as possible by another blind researcher. The principal investigator (MC) will not be blinded and will respond to any clinical or research issues during the trial that require knowledge of a participants’ group allocation.

### Procedure for unblinding if needed {17b}

There are no circumstances under which unblinding of researchers conducting the T1 and T2 assessments is permissible.

## Data collection and management

### Plans for assessment and collection of outcomes {18a}

Table 1 gives an overview about the diagnostic interviews and questionnaires and the assessment schedule.

**Table 1:** Assessment schedule

		T0	T1	T2
Interviews	Demographic information	x		
	Characteristics of the TAU	x	x	x
	Structured Interview for Psychosis-Risk Syndromes (SIPS)	x		
	Structured Clinical Interviews for Mental Disorders (DIPS)/Structured Clinical Interview for Mental Disorders in children and adolescents (Kinder-DIPS)	x		
	Module B (psychotic disorders) of the Structured Clinical Interview for DSM-5 Disorders—Clinician Version (SCID-5-CV)	x		
	BPD module of the Structured Clinical Interview for DSM-5 Personality Disorders (SCID-5-PD)	x		
	Psychotic Symptom Rating Scales Auditory Hallucinations subscale (PSYRATS-AH)	x	x	x
	Clinical Global Impression Scale-Severity (CGIs)	x	x	x
	Social and Occupational Functioning Assessment Scale (SOFAS)	x	x	x
Questionnaires	International Trauma Questionnaire (ITQ)/International Trauma Questionnaire—Child and Adolescent version (ITQ-CA)	x		
	Child and Adolescent Trauma Screen (CATS)	x		
	Childhood Trauma Questionnaire (CTQ)	x		
	Dissociation Tension Scale (DSS)	x		
	Revised Beliefs About Voices Questionnaires (BAVQ-R)	x	x	x
	Voice and You—Revised (VAY-R)	x	x	x
	Approve questionnaires	x	x	x
	Brief Core Schema Scales (BCSS)	x	x	x
	Depression, Anxiety and Stress Scale-21 (DASS-21)	x	x	x
	Quality of Life Enjoyment and Satisfaction Questionnaire (Q-LES-Q-18)	x	x	x
	Treatment satisfaction questionnaire		x	

#### Eligibility screening

The eligibility screening is informed by the PSYRATS-AH [30]. A description of the measure is given below.

#### Measures to characterize the sample

##### Sociodemographic information

This interview assesses sociodemographic variables such as age, gender, current education or employment, living situation, relationship status, and income.

##### Clinical information

Detailed information on psychiatric disorders in first-degree relatives, the treatment history, and the type and extent of the TAU will be obtained in a structured way.

##### Structured interview for Psychosis-Risk Syndromes (SIPS) [54]

The SIPS is a commonly used instrument to assess clinical high-risk (CHR) syndromes for psychosis. The SIPS includes a 19-item severity scale, the Scale Of Psychosis-risk Symptoms (SOPS), that measures five positive, six negative, four disorganization, and four general symptoms, each scored from 0 (absent) to 6 (severe and psychotic). Participants meet threshold for a SIPS-defined risk syndrome or a first-episode psychosis based on their score on one or more positive symptoms. High predictive validity and interrater reliability have been reported [55].

##### Diagnostic Interview for Mental Disorders (Diagnostisches Interview bei Psychischen Störungen; DIPS) [56]/Diagnostic Interview for Mental Disorders in Children and Adolescents (Diagnostisches Interview bei Psychischen Störungen im Kindes- und Jugendalter; Kinder-DIPS) [57]

The DIPS interviews are structured clinical interviews to assess mental disorders in adults (≥ 16 years of age) and children/adolescents (6–18 years of age), respectively, according to the ICD-10 and DSM-5. While the Kinder-DIPS includes two parallel versions for children/adolescents and their parents, only the former one will be applied in the current trial. Adequate validity and reliability have been reported for both the DIPS [58, 59] and the Kinder-DIPS [60–62].

##### Module B (psychotic disorders) of the Structured Clinical Interview for DSM-5 Disorders—Clinician Version (SCID-5-CV) [63]

Since the DIPS interviews contain only a brief screener for psychotic disorders, module B of the SCID-5-CV is applied to assess psychotic disorders according to the ICD-10 and DSM-5.

##### Borderline personality disorder (BPD) module of the Structured Clinical Interview for Personality Disorders (SCID-5-PD) [64]

The SCID-V-PD is a structured clinical interview for the detection of personality disorders according to the DSM-5. In the current trial, the BPD module will be administered as there is increasing evidence suggesting that hearing voices is a common phenomenon in individuals with BPD [65]. The BPD module examines the presence of the nine criteria for BPD defined by DSM-5. Each item is scored on a three-point scale (1=absent, 2=subthreshold, 3=present). The diagnosis requires that five or more criteria are met. A BPD criterion is scored as met if it has been present for 2 years for individuals older than 18 years, and for 1 year for under-age individuals [66]. The BPD module of the SCID-V-PD has been widely used in clinical and research settings for adults and has also been successfully adopted for the adolescent population [67].

##### International Trauma Questionnaire (ITQ) [68]/International Trauma Questionnaire—Child and Adolescent version (ITQ-CA) [69]

The ITQ and ITQ-CA are valid and reliable measures of ICD-11 posttraumatic stress disorder (PTSD) and Complex PTSD (CPTSD) for adults and children/adolescents (aged 7 to 17), respectively. They are included in the current trial, as evidence indicates that hearing voices is a common phenomenon in patients with trauma-related disorders [70, 71]. The ITQ measures will be used in conjunction with the traumatic events checklist of the *Child and Adolescent Trauma Screen (CATS)* [72]. The list includes 14 potentially traumatic experiences and participants are asked to indicate if they experienced any of those, using a binary “yes/no” response. If a participant experienced at least one traumatic event, the CATS will be followed by the ITQ or the ITQ-CA, respectively. The ITQ measures include 12 items reflecting symptoms of PTSD (i.e., re-experiencing, avoidance, sense of threat) and disturbances in self-organization (i.e., affective dysregulation, negative self-concept, disturbances in relationships). Participants indicate on a 5-point Likert-Scale ranging from 0 (never) to 4 (almost always) how much they have been bothered by each symptom over the past month. Scores of ≥ 2 (moderately) indicate the presence of a symptom. Additionally, five items assess functional impairment in different areas of life (e.g., friends, family, school, hobbies), using a binary yes/no scale. The presence of at least one symptom from each PTSD cluster and at least one indicator of functional impairment is required for a PTSD diagnosis. The presence of at least one symptom from each PTSD and disturbances in the self-organization cluster and at least one indicator of functional impairment is required for a CPTSD diagnosis. If participants meet the criteria for CPTSD, the PTSD diagnosis is excluded.

##### Childhood Trauma Questionnaire (CTQ) [73]

The CTQ is a 28-item self-report retrospective inventory to measure childhood or adolescent emotional, physical, and sexual abuse, and emotional and physical neglect. A 5-point scale is used, ranging from 1 (never true) to 5 (very often true). The psychometric properties of the German version are similar to the American original [74].

##### Dissociation Tension Scale (DSS) [75]

The DSS is a self-rating instrument for the assessment of psychological and somatoform dissociative features (ranging from normal up to pathological) as well as of aversive inner tension, occurring within the past 7 days. The DSS contains 21 items, rated on a time-oriented scale ranging from 0% (never) to 100% (constantly).

#### Primary outcome measure

##### Distress dimension of the Psychotic Symptom Rating Scales—Auditory Hallucination subscale (PSYRATS-AH) [30]

The PSYRATS-AH is a structured interview examining 11 dimensions of auditory hallucinations (i.e., frequency, duration, location, loudness, beliefs regarding the origin, amount of negative content, degree of negative content, amount of distress, intensity of distress, disruption, control), each rated on a 5-point scale, with higher scores representing greater overall severity of voice hearing experiences. Items are grouped in four subscales measuring distress (negative content, distress, and control), frequency (frequency, duration, disruption), attribution (location and origin of voices), and loudness (loudness item only) [76]. The German version achieved good reliability and validity scores [77].

#### Secondary outcome measures

##### Revised Beliefs About Voices Questionnaire (BAVQ-R) [78]

The persecutory beliefs subscale [79] of the BAVQ-R will be used in the current study to assess perceived hostile intent and power of voices. The BAVQ-R is a self-report measure of cognitive, emotional, and behavioral responses to voices. It consists of 35 items rated on a 4-point Likert scale from 0 (disagree) to 3 (strongly agree). The original measure included five subscales, three relating to beliefs about voices (i.e., malevolence, benevolence, and omnipotence) and two relating to emotional and behavioral responses to voices (i.e., engagement and resistance). For the German version, the beliefs subscales with the exception of the omnipotence subscale showed satisfying internal consistency and good test-retest-reliability [38]. A recent study examining the factor structure of the BAVQ-R supported a two-factor (persecutory beliefs combining omnipotence and malevolence, and benevolent beliefs) instead of the proposed three-factor beliefs model [79].

##### Voice and You-Revised (VAY-R) [39]

The voice dominance and intrusiveness subscales of the VAY-R will be used to assess dysfunctional beliefs about voices. The VAY-R is a brief version of the VAY [80] measuring both the extent of a perceived aggressive relating of the voice in terms of dominance and intrusiveness and the extent of reciprocal submissive (i.e., hearer dependence) and avoidant (i.e., hearer distance) relating preferences of the voice-hearer. It consists of 14 items, measured on a 4-point scale ranging from 0 (seldom) to 3 (nearly always). The VAY has good internal consistency and acceptable test-retest reliability [80]. The psychometric properties of the German version are currently under examination by Lincoln et al. [37].

##### Approve questionnaires [40]

The Approve-Voices and the Approve-Social measures each consist of 15 items measuring functional assertive and dysfunctional non-assertive (i.e., passive or aggressive) relating to voices and to other people, respectively. Items are scored on a 10-point Likert scale, ranging from 0 (disagree completely) to 10 (agree completely). The Approve questionnaires have been found to be reliable and valid in both their English [40] and German versions, with the German validation study being currently in preparation for publication [37].

##### Brief Core Schema Scales (BCSS) [41]

The BCSS is a self-report measure of schemata concerning self and others in psychosis with good psychometric properties. The 24 items are rated on a 5-point Likert scale from 0 (I do not hold the belief) to 4 (I believe it totally) and form four subscales, assessing negative self, positive self, negative other, and positive other.

#### Other outcome measures

##### Depression, Anxiety and Stress Scale-21 (DASS-21) [81]

The 21-item version of the DASS-21 will be administered to assess for features of depression (depression subscale), hyperarousal (anxiety subscale), and tension (stress subscale) over the previous week. The items are rated on a 4-point Likert scale from 0 (did not apply to me at all) to 3 (applied to me very much, or most of the time). Psychometric properties have been established in clinical and community samples [82–84] for the original version as well as for the German version [43].

##### Clinical Global Impression Scale—Severity (CGIs) [44]

The CGIs is an observer-rated global measure of symptom severity within the past 7 days that has been widely used in clinical trials. It ranges from 1 (not ill at all) to 7 (severely ill).

##### Social and Occupational Functioning Assessment Scale (SOFAS) [45]

The SOFAS is an observer-rated measure of the individual’s level of social and educational/occupational functioning, independently of the severity of the individual’s psychopathology. The scale ranges from 0 to 100, with higher scores indicating better functioning.

##### Quality of Life Enjoyment and Satisfaction Questionnaire (Q-LES-Q-18) [46]

The Q-LES-Q18 is a self-report questionnaire assessing life satisfaction in five dimensions: physical health, subjective feelings, leisure activities, social relationships, and medication. Its 18 items are rated on a 5-point scale, with (1) denoting “not at all” and (5) “all the time.”

##### Treatment satisfaction questionnaire

A short questionnaire was developed for the current study to assess satisfaction with the study procedure and the CBTv-based EMI under examination. The items are rated on a 7-point Likert ranging from 1 (not true at all) to 7 (completely true).

Researchers will receive training in the administration of the clinical interviews before they start conducting the assessments. Additionally, the maintenance of high-quality diagnostic assessments will be facilitated by continuous supervision.

#### EMA

The two EMA periods will last 9 days each and have a time-contingent design with a semi-random sampling schedule. Participants will receive ten text messages a day between 8:00am and 10:30pm, prompting them to complete an EMA survey. Each prompt will take place randomly within equal intervals of 60 min, with 30 min between intervals in order to prevent prompts occurring too close together. Each EMA survey takes approximately 2–3 min to complete and assesses sleeping problems (only in the first survey of the day); affective state; self-esteem; the occurrence of voices since the previous assessment; perceived distress of voices; beliefs about voices; passive, aggressive, and assertive relating to voices; social contact since the last assessment; passive, aggressive, and assertive relating to other people; and dissociative experiences, traumatic memories, non-suicidal self-injury, suicidal thoughts, and alcohol and drug consumption since the last assessment. A full list of the EMA items is provided in Additional file 1.

Before the baseline EMA, participants will be instructed how to use the EMA and practice its usage by going through a survey to ensure that each item is understood and appropriately rated. They will be given a written confirmation of participation in the study, which can be presented to teachers at school or supervisors at work if required. Participants will be encouraged to contact the research team immediately if they have any questions or technical issues related to the EMA.

### Plans to promote participant retention and complete follow-up {18b}

Efforts will be made to engage participants in as many assessments as possible and achieve a high adherence rate in the EMA. Appointments for the assessments will be offered at times and locations, which best suit the participants. Additionally, during assessments, breaks will be offered and the assessments can be split into serval shorter sessions if required. Participants adherence to the assessment schedules of the two EMA periods and the EMI will be monitored electronically by the research team, using the administration tool of the web-based software program ASMO. Participants showing a significant drop in their response rate will be contacted by the research team to clarify the reasons of non-adherence, boost motivation, and offer problem-solving and support when needed. Participants will be reimbursed for participation in the baseline assessment (T0) with 75 CHF, and for the participation in the 3-month (T1) and 6-month (T2) follow-up assessments with 25 CHF each. Additionally, they will get reimbursed for each completed survey of the two EMA periods with 0.50 CHF (90 CHF in total) and with additionally 10 CHF for a completion rate of ≥ 70%. Retention rates will be continuously monitored by the principal investigator throughout the trial.

### Data management {19}

Diagnostic interview and self-report data assessed at baseline (T0), 3-month follow-up (T1), and 6-month follow-up (T2) will be collected in encrypted form and managed using REDCap (Research Electronic Data Capture) tools hosted at the University Bern, Switzerland [51, 52]. REDCap is a secure, web-based software platform designed to support data capture for research studies, providing (1) an intuitive interface for validated data capture; (2) audit trails for tracking data manipulation and export procedures; and (3) automated export procedures for seamless data downloads to common statistical packages. The exported data will be stored on the end-to-end encrypted cloud storage system (Tresorit: www.tresorit.com) of the research department of the University Hospital of Child and Adolescent Psychiatry and Psychotherapy Bern, University of Bern, Switzerland. The storage system is regularly backed up and checked by the biostatistician (SL) of the trial.

The EMA/I data collected via the web-based software program ASMO will be stored in encrypted form on secure servers, located at the Centre for Psychotherapy Research, University of Heidelberg, Germany. A distributed Replicated Block Devide (DRBD)-based cluster will provide synchronous replication of all data during data entry on two separate servers. In addition, incremental backups will be conducted following a predefined schedule. The EMA/I will be transferred via an encrypted, password-protected data exchange portal located at the server of the Centre for Psychotherapy Research, to the end-to-end encrypted cloud storage system Tresorit of the research department of the University Hospital for Child and Adolescent Psychiatry and Psychotherapy, University of Bern, Switzerland. Afterwards, the EMA/I data stored in Heidelberg will be deleted.

All members of the research team involved in the collection and management of data will be given training on how to use the web-based software programs REDCap and ASMO. The researchers involved in the day-to-day data collection will be supervised by the principal investigator (MC) and the biostatistician (SL) of the trial. Data collection and management will be a standing item on the agenda of the weekly meeting of the research team.

### Confidentiality {27}

All data collected within the trial will be kept confidential and be handled with uttermost discretion. Confidentiality will only be broken if participants disclose any information that indicates a risk for themselves or others. Data collection via REDCap and ASMO will occur in encrypted form only. The coding key linking a unique participant number with personal data of participants will be stored in a password-protected file on the end-to-end encrypted cloud storage system Tresorit of the research department of the University Hospital for Child and Adolescent Psychiatry and Psychotherapy, University of Bern, Switzerland. Physical data, such as consent forms, will be locked in filing cabinets of the research department of the University Hospital for Child and Adolescent Psychiatry and Psychotherapy, University of Bern, Switzerland. The coding key, the consent forms, and all used electronic systems (i.e., REDCap, ASMO, Tresorit) will be only accessible to authorized personnel who fulfill their duties within the scope of the trial. Further, the electronic systems have built-in audit trails that log all user activity. The data storage will be continuously monitored by the principal investigator (MC) and the biostatistician (SL) of the trial. Data will be analyzed and published in aggregated and encrypted form only.

### Plans for collection, laboratory evaluation, and storage of biological specimens for genetic or molecular analysis in this trial/future use {33}

Not applicable—this trial does not involve collecting biological specimens.

## Statistical methods

### Statistical methods for primary and secondary outcomes {20a}

Descriptive statistics within each randomized group will be presented for all variables assessed at baseline (T0), 3-month follow-up (T1), and 6-month follow-up (T2). To examine the effect of CBTv-based EMI on the primary outcome of voice distress (PSYRATS-AH Distress), multilevel regression models will be applied, with the participants’ ID as random, as well as group allocation (TAU + CBTv-based EMI versus TAU), time (T0, T1, T2), and PSYRATS-AH Distress at baseline as fixed effects. Multilevel modeling is advantageous over traditional methods of repeated-measures analysis, as it uses all available data and is unaffected by randomly missing data (135). The same approach will be used to analyze the effects of the CBTv-based EMI on secondary and other pre-specified outcomes. All analyses will be run based on the intention-to-treat principal (ITT) and be adjusted for confounding variables (e.g., sex, age). Standardized effect sizes will be reported. If needed, significance level adjustment will be applied to prevent accumulation of the type I error. Statistical analyses will be carried out using Stata Statistical Software [53] or R [85].

### Interim analyses {21b}

No interim analyses planned.

### Methods for additional analyses (e.g., subgroup analyses) {20b}

In order to shed some light on potential mechanisms of change underlying the proposed efficacy of the CBTv-based EMI, appraisals of and relating styles to voices as well as core beliefs about the self will be examined as potential mediators [35, 42]. For this purpose, time-lagged multilevel network analysis will be conducted on data of the two EMA periods. Network analysis allows for the examination and visualization of changes in the temporal interrelationships between variables [86]. Changes in network connectivity between the networks of the two EMA periods will be compared between the two treatment conditions.

In order to shed some light on the question of who benefits from the CBTv-based EMI and who does not, potential moderators (e.g., diagnostic group (psychotic vs. non-psychotic disorders), continuous vs. non-continuous voices) will be explored. This will be done by repeating the primary multilevel regression models and including interaction terms between the group and each moderator variable. The interaction term indicates whether the treatment effect is different at different levels of the moderator variable.

### Methods in analysis to handle protocol non-adherence and any statistical methods to handle missing data {20c}

All statistical analyses will be run based on the ITT principal where all participants who are randomized are included in the analyses according to their group allocation, regardless of whether they completed the study or terminated it early. Since multilevel models can handle missing observations due to dropout (assuming data are missing at random), imputation of missing values will not be needed.

### Plans to give access to the full protocol, participant-level data, and statistical code {31c}

In line with the open research data principals of the Swiss National Science Foundation (SNSF), the data and statistical codes related to publications will be made directly and freely available in a data repository that meets the FAIR criteria [87]. Data will be shared in encrypted form only. The possibility of data sharing will be made explicit to participants on the consent form.

## Oversight and monitoring

### Composition of the coordinating center and trial steering committee {5d}

The coordinating center is the research department of the University Hospital for Child and Adolescent Psychiatry and Psychotherapy, University of Bern, Switzerland (MC, JK, SL, MK). It will be responsible for running the trial day-to-day, including the recruitment and data collection and management. MH will provide supervision regarding the EMI introduction and closing sessions.

The Trials Steering Committee (TSC) includes an independent chair, two independent experts, the PI (MC), and the director of the University Hospital for Child and Adolescent Psychiatry and Psychotherapy, University of Bern, Switzerland (MK). It will oversee the scientific integrity of the trial, including patient safety, progress of the trial, and adherence to protocols.

### Composition of the data monitoring committee, its role, and reporting structure {21a}

There will be no data monitoring committee. Monitoring will be done by the biostatistician of the University Hospital for Child and Adolescent Psychiatry and Psychotherapy, University of Bern (SL), who is independent of the research team. There will be an initial monitoring before the start of the study to ensure that all data collection systems are correctly set up. Afterwards, there will be a brief monitoring every month to ensure adherence to procedures and an extensive monitoring every 6 months to spot-check the proper data collection and storage and the integrity of the data collected.

### Adverse event reporting and harms {22}

An adverse event (AE) is defined as any untoward medical occurrence in a participant which does not necessarily have a causal relationship with the trial procedure. A serious adverse event (SAE) is any untoward medical occurrence that (a) results in death or is life-threatening, (b) requires in-patient hospitalization or prolongation of existing hospitalization, (c) results in persistent or significant disability or incapacity, or (d) causes a congenital anomaly or birth defect. The principal investigator (MC) will make a causality assessment of the event to the trial intervention. Any event assessed as possibly, probably, or definitely related is classified as related to the trial intervention. Additionally, the principal investigator (MC) makes a severity assessment of the event as mild, moderate, or severe. Mild means the complication is tolerable, moderate means it interferes with daily activities, and severe means it renders daily activities impossible. All SAEs are documented and reported immediately (within a maximum of 24 h) to principal investigator (MC) and the director of the University Hospital for Child and Adolescent Psychiatry and Psychotherapy, University of Bern, Switzerland (MK). If it cannot be excluded that the SAE is attributable to the intervention under investigation, the principal investigator (MC) reports it to the Ethics Committee of the Canton Bern, Switzerland, within 15 days. The TSC will be informed of SAE periodically.

### Frequency and plans for auditing trial conduct {23}

The Ethics Committee of the Canton Bern, Switzerland, can visit the research sites for a trial auditing. Direct access to the source data and all study-related files is granted on such occasions. All involved parties keep the participant data strictly confidential.

### Plans for communicating important protocol amendments to relevant parties (e.g., trial participants, ethical committees) {25}

Substantial amendments are defined as changes that affect the safety, health, rights and obligations of participants; changes in the protocol that affect study objective(s) or central research topic; and changes of study site(s) or of study leader and sponsor. They will be submitted to the Ethics Committee of the Canton Bern, Switzerland, for approval before implementation. Under emergency circumstances, deviations from the protocol to protect the rights, safety, and well-being of participants may proceed without prior approval of the Ethics Committee. Such deviations will be documented and reported to the Ethics Committee as soon as possible. A list of all non-substantial amendments will be submitted once a year to the Ethics Committee. Additionally, substantial protocol modifications will be communicated to the trial registries.

### Dissemination plans {31a}

We intend to make the findings of the trial accessible to other researchers, clinicians, young people affected by distressing voices and their families, and other interested people from the general population. Therefore, the findings will be disseminated through publications in open-access scientific journals, the presentation at key scientific conferences of the field as well as at public events on mental health in young people, and publications on social media and relevant websites addressing the experience of voice hearing or mental health in youth.

## Discussion

This is the first study to examine the efficacy of CBTv, delivered using smartphone-based EMI in a transdiagnostic sample of youth with distressing voices. The study helps to bridge several clinical and scientific gaps. Clinically, it provides a highly specialized, low-intensity and flexible treatment opportunity for a hitherto neglected patient group for whom specialized treatment has been scarce and who are known for their low engagement with conventional mental health services. Thus, the trial has the potential to make a significant contribution to early intervention in order to prevent negative long-term outcomes and improve the life trajectories of the affected young people. Scientifically, the study will contribute to clarify whether CBTv is suitable for young voice-hearers and for voice-hearers without a psychotic disorder, whether CBTv can be successfully delivered using smartphone-based EMI, what the mechanisms of change through therapy are, and who benefits from therapy and who does not.

Despite these benefits, the following limitations of the study design have to be acknowledged. First, the EMI is based on CBTv that was recently developed for adults with distressing voices and has been evaluated in patients with primarily psychotic disorders so far. Any change to an existing therapy in terms of content, format, or target group involves the risk that it is no longer or less effective. Second, the definition of TAU as including both receiving no psychiatric/psychological treatment at all and any form of psychiatric/psychological was chosen to make the intervention accessible to as many young people as possible, but creates the disadvantage of the TAU being heterogeneous. Third, reaching the target number of 154 participants for the trial may be challenging. However, the risk of low recruitment numbers is addressed through the multicenter approach. Finally, e-mental health interventions are less obliging than traditional face-to-face treatments and may therefore suffer from lower adherence and higher drop-out rates. The research team will pay special attention to keeping participants in the study. In addition, participants will be overrecruited to compensate for a dropout rate of up to 20%.

If the present study is successful despite the discussed potential challenges, it may make a significant contribution to early intervention for young people with distressing voices.

## Trial status

The study protocol was approved by the Ethics Committee of the Canton Bern, Switzerland, on the 19 of August 2021 (protocol version 1.2). Recruitment is scheduled to start in autumn 2022 and is expected to last up to 4 years.

## Supplementary Information

**Additional file 1.** Smartphone-based EMA items. Smartphone-based EMI items.

## Data Availability

In line with the open research data principals of the SNSF, the data and statistical codes related to publications will be made directly and freely available in a data repository that meets the FAIR criteria [87]. Data will be shared in encrypted form only. The possibility of data sharing will be made explicit to participants on the consent form.

## Abbreviations

AVH: Auditory verbal hallucinations
BAVQ-R: Revised Beliefs About Voices Questionnaire
BCSS: Brief Core Schema Scales
BPD: Borderline Personality Disorder
CATS: Child and Adolescents Trauma Screen
CBTv: Cognitive Behavioral Therapy for voices
CBTp: Cognitive Behavioral Therapy for psychosis
CGIs: Clinical Global Impression Scale-Severity
CHF: Swiss Francs
CONSORT: Consolidated Standards of Reporting Trials
CTQ: Childhood Trauma Questionnaire
DASS-21: Depression, Anxiety and Stress Scale-21
DIPS: Diagnostisches Interview für psychiche Störungen (engl., Diagnostic Interview for Mental Disorders)
DSS: Dissociation Tension Scale
EMA: Ecological Momentary Assessment
EMI: Ecological Momentary Intervention
ITQ: International Trauma Questionnaire
ITQ-CA: International Trauma Questionnaire—Child and Adolescent version
ITT: Intention To Treat
Kinder-DIPS: Diagnostisches Interview für psychiatrische Störungen bei Kindern und Jugendlichen (engl. Diagnostic Interview for Mental Disorders in Children and Adolescents)
Q-LES_Q-18: Quality of Life Enjoyment and Satisfaction Questionnaire
PSYRATS-AH: Psychotic Symptom Rating Scales Auditory Hallucinations subscale
RCT: Randomized Controlled Trial
REDCap: Research Electronic Data Capture
SCID-5-PD: Structured Clinical Interview for DSM-5 Personality Disorders
SIPS: Structured Interview for Psychosis-Risk Syndromes
SmartVocies: Smartphone-assisted guided self-help cognitive behavioral therapy for young people with distressing voices
SMS: Short Message Service
SNSF: Swiss National Science Foundation
SOFAS: Social and Occupational Functioning Assessment Scale
TAU: Treatment As Usual
TSC: Trial Steering Committee

## References

[1] Waters F, Allen P, Aleman A, Fernyhough C, Woodward TS, Badcock JC (2012). Auditory hallucinations in schizophrenia and nonschizophrenia populations: a review and integrated model of cognitive mechanisms. Schizophr Bull.

[2] Baumeister D, Sedgwick O, Howes O, Peters E (2017). Auditory verbal hallucinations and continuum models of psychosis: a systematic review of the healthy voice-hearer literature. Clin Psychol Rev.

[3] Maijer K, Begemann MJH, Palmen SJMC, Leucht S, Sommer IEC (2018). Auditory hallucinations across the lifespan: a systematic review and meta-analysis. Psychol Med.

[4] Rubio JM, Sanjuán J, Flórez-Salamanca L, Cuesta MJ (2012). Examining the course of hallucinatory experiences in children and adolescents: a systematic review. Schizophr Res.

[5] Kelleher I, Devlin N, Wigman JTW, Kehoe A, Murtagh A, Fitzpatrick C (2014). Psychotic experiences in a mental health clinic sample: implications for suicidality, multimorbidity and functioning. Psychol Med.

[6] Bartels-Velthuis AA, Wigman JTW, Jenner JA, Bruggeman R, van Os J (2016). Course of auditory vocal hallucinations in childhood: 11-year follow-up study. Acta Psychiatr Scand.

[7] De Loore E, Gunther N, Drukker M, Feron F, Sabbe B, Deboutte D (2011). Persistence and outcome of auditory hallucinations in adolescence: a longitudinal general population study of 1800 individuals. Schizophr Res.

[8] Fujita J, Takahashi Y, Nishida A, Okumura Y, Ando S, Kawano M (2015). Auditory verbal hallucinations increase the risk for suicide attempts in adolescents with suicidal ideation. Schizophr Res.

[9] Poulton R, Caspi A, Moffitt TE, Cannon M, Murray R, Harrington H (2000). Children’s self-reported psychotic symptoms and adult schizophreniform disorder: a 15-year longitudinal study. Arch Gen Psychiatry.

[10] Maijer K, Palmen SJMC, Sommer IEC (2017). Children seeking help for auditory verbal hallucinations; who are they?. Schizophr Res.

[11] Thomas N, Hayward M, Peters E, van der Gaag M, Bentall RP, Jenner J (2014). Psychological therapies for auditory hallucinations (voices): current status and key directions for future research. Schizophr Bull.

[12] Mawson A, Cohen K, Berry K (2010). Reviewing evidence for the cognitive model of auditory hallucinations: the relationship between cognitive voice appraisals and distress during psychosis. Clin Psychol Rev.

[13] Hayward M, Berry K, Ashton A (2011). Applying interpersonal theories to the understanding of and therapy for auditory hallucinations: a review of the literature and directions for further research. Clin Psychol Rev.

[14] Birchwood M, Meaden A, Trower P, Gilbert P, Plaistow J (2000). The power and omnipotence of voices: subordination and entrapment by voices and significant others. Psychol Med.

[15] Birchwood M, Gilbert P, Gilbert J, Trower P, Meaden A, Hay J (2004). Interpersonal and role-related schema influence the relationship with the dominant ‘voice’ in schizophrenia: a comparison of three models. Psychol Med.

[16] Hayward M (2003). Interpersonal relating and voice hearing: to what extent does relating to the voice reflect social relating?. Psychol Psychother.

[17] Lincoln TM, Peters E (2019). A systematic review and discussion of symptom specific cognitive behavioural approaches to delusions and hallucinations. Schizophr Res.

[18] Hazell CM, Greenwood K, Fielding-Smith S, Rammou A, Bogen-Johnston L, Berry C (2018). Understanding the barriers to accessing symptom-specific cognitive behavior therapy (CBT) for distressing voices: reflecting on and extending the lessons learnt from the CBT for psychosis literature. Front Psychol.

[19] Gulliver A, Griffiths KM, Christensen H (2010). Perceived barriers and facilitators to mental health help-seeking in young people: a systematic review. BMC Psychiatry.

[20] McGorry PD, Bates T, Birchwood M (2013). Designing youth mental health services for the 21st century: examples from Australia, Ireland and the UK. Br J Psychiatry.

[21] Lambert M, Bock T, Naber D, Löwe B, Schulte-Markwort M, Schäfer I (2013). Die psychische Gesundheit von Kindern, Jugendlichen und jungen Erwachsenen – Teil 1: Häufigkeit, Störungspersistenz, Belastungsfaktoren, Service-Inanspruchnahme und Behandlungsverzögerung mit Konsequenzen. Fortschr Neurol Psychiatr.

[22] Dixon LB, Holoshitz Y, Nossel I (2016). Treatment engagement of individuals experiencing mental illness: review and update: •••. World Psychiatry.

[23] Patel V, Flisher AJ, Hetrick S, McGorry P (2007). Mental health of young people: a global public-health challenge. Lancet.

[24] Nahum-Shani I, Smith SN, Spring BJ, Collins LM, Witkiewitz K, Tewari A (2018). Just-in-time adaptive interventions (JITAIs) in mobile health: key components and design principles for ongoing health behavior support. Ann Behav Med.

[25] Seko Y, Kidd S, Wiljer D, McKenzie K (2014). Youth mental health interventions via mobile phones: a scoping review. Cyberpsychol Behav Soc Netw.

[26] Kenny R, Dooley B, Fitzgerald A (2016). Developing mental health mobile apps: exploring adolescents’ perspectives. Health Informatics J.

[27] Reininghaus U, Depp CA, Myin-Germeys I (2016). Ecological interventionist causal models in psychosis: targeting psychological mechanisms in daily life. Schizophr Bull.

[28] Bell IH, Lim MH, Rossell SL, Thomas N (2017). Ecological momentary assessment and intervention in the treatment of psychotic disorders: a systematic review. Psychiatr Serv.

[29] Bell IH, Rossell SL, Farhall J, Hayward M, Lim MH, Fielding-Smith SF (2020). Pilot randomised controlled trial of a brief coping-focused intervention for hearing voices blended with smartphone-based ecological momentary assessment and intervention (SAVVy): Feasibility, acceptability and preliminary clinical outcomes. Schizophr Res.

[30] Haddock G, McCarron J, Tarrier N, Faragher EB (1999). Scales to measure dimensions of hallucinations and delusions: the psychotic symptom rating scales (PSYRATS). Psychol Med.

[31] Bauer S, Moessner M (2012). Technology-enhanced monitoring in psychotherapy and e-mental health. J Ment Health.

[32] Kaess M, Koenig J, Bauer S, Moessner M, Fischer-Waldschmidt G, the STAR Consortium (2019). Self-injury: Treatment, Assessment, Recovery (STAR): online intervention for adolescent non-suicidal self-injury - study protocol for a randomized controlled trial. Trials.

[33] Kaess M, Bauer S (2019). Editorial promoting help-seeking using E-Technology for adolescents: the ProHEAD consortium. Trials.

[34] Hayward M, Strauss C, Kingdon D (2018). Overcoming distressing voices.

[35] Hayward M, Jones A-M, Bogen-Johnston L, Thomas N, Strauss C (2017). Relating therapy for distressing auditory hallucinations: a pilot randomized controlled trial. Schizophr Res.

[36] Craig TK, Rus-Calafell M, Ward T, Leff JP, Huckvale M, Howarth E (2018). AVATAR therapy for auditory verbal hallucinations in people with psychosis: a single-blind, randomised controlled trial. Lancet Psychiatry.

[37] Lincoln TM, Pillny M, Schlier B, Hayward M (2021). RELATE—a randomised controlled feasibility trial of a Relating Therapy module for distressing auditory verbal hallucinations: a study protocol. BMJ Open.

[38] Gmeiner A, Aslan J, Gaglia A, Rumpold T, Schrank B, Süßenbacher S (2018). Überzeugungen und Belastungen durch Stimmenhören: die deutsche Version des Beliefs About Voices Questionnaire – Revised (BAVQ-R). Neuropsychiatrie.

[39] Schlier B, Strauss C, Lincoln TM, Hayward M (2020). Relating between the voice and voice-hearer: validation of a revised version of the Voice And You. Schizophr Res.

[40] Hayward M, Schlier B, Strauss C, Rammou A, Lincoln T (2020). Construction and validation of the Approve questionnaires - measures of relating to voices and other people. Schizophr Res.

[41] Fowler D, Freeman D, Smith B, Kuipers E, Bebbington P, Bashforth H (2006). The Brief Core Schema Scales (BCSS): psychometric properties and associations with paranoia and grandiosity in non-clinical and psychosis samples. Psychol Med.

[42] Hazell CM, Hayward M, Cavanagh K, Jones A-M, Strauss C (2018). Guided self-help cognitive-behaviour Intervention for VoicEs (GiVE): results from a pilot randomised controlled trial in a transdiagnostic sample. Schizophr Res.

[43] Nilges P, Essau C (2015). Die Depressions-Angst-Stress-Skalen: Der DASS – ein Screeningverfahren nicht nur für Schmerzpatienten. Schmerz.

[44] Guy W (1976). ECDEU assessment manual for psychopharmacology.

[45] Goldman HH, Skodol AE, Lave TR (1992). Revising axis V for DSM-IV: a review of measures of social functioning. Am J Psychiatry.

[46] Ritsner M, Kurs R, Gibel A, Ratner Y, Endicott J (2005). Validity of an abbreviated quality of life enjoyment and satisfaction questionnaire (Q-LES-Q-18) for schizophrenia, schizoaffective, and mood disorder patients. Qual Life Res.

[47] Chan A-W, Tetzlaff JM, Altman DG, Laupacis A, Gøtzsche PC, Krleža-Jerić K (2013). SPIRIT 2013 statement: defining standard protocol items for clinical trials. Ann Intern Med.

[48] Faul F, Erdfelder E, Lang A-G, Buchner A (2007). G*Power 3: a flexible statistical power analysis program for the social, behavioral, and biomedical sciences. Behav Res Methods.

[49] Scott NW, McPherson GC, Ramsay CR, Campbell MK (2002). The method of minimization for allocation to clinical trials. a review. Control Clin Trials.

[50] Pocock SJ, Simon R (1975). Sequential treatment assignment with balancing for prognostic factors in the controlled clinical trial. Biometrics.

[51] Harris PA, Taylor R, Thielke R, Payne J, Gonzalez N, Conde JG (2009). Research electronic data capture (REDCap)—a metadata-driven methodology and workflow process for providing translational research informatics support. J Biomed Inform.

[52] Harris PA, Taylor R, Minor BL, Elliott V, Fernandez M, O’Neal L (2019). The REDCap consortium: building an international community of software platform partners. J Biomed Inform.

[53] StataCorp (2019). Stata statistical software. Release 16.

[54] McGlashan T, Walsh B, Woods S (2010). The psychosis-risk syndrome: handbook for diagnosis and follow-up.

[55] Miller TJ, McGlashan TH, Rosen JL, Cadenhead K, Ventura J, McFarlane W (2003). Prodromal assessment with the structured interview for prodromal syndromes and the scale of prodromal symptoms: predictive validity, interrater reliability, and training to reliability. Schizophr Bull.

[56] Margraf J, Cwik JC, Pflug V, Schneider S (2017). Strukturierte klinische Interviews zur Erfassung psychischer Störungen über die Lebensspanne: Gütekriterien und Weiterentwicklungen der DIPS-Verfahren. Z Klin Psychol Psychother.

[57] Schneider S, Pflug V, Margraf J, In-Albon T (2017). Kinder-DIPS: Diagnostisches Interview bei psychischen Störungen im Kindes- und Jugendalter.

[58] In-Albon T, Suppiger A, Schlup B, Wendler S, Margraf J, Schneider S (2008). Validität des Diagnostischen Interviews bei psychischen Störungen (DIPS für DSM-IV-TR). Z Klin Psychol Psychother.

[59] Suppiger A, In-Albon T, Herren C, Bader K, Schneider S, Margraf J (2008). Reliabilität des Diagnostischen Interviews bei Psychischen Störungen (DIPS für DSM-IV-TR) unter klinischen Routinebedingungen. Verhaltenstherapie.

[60] Popp L, Neuschwander M, Mannstadt S, In-Albon T, Schneider S. Parent-child diagnostic agreement on anxiety symptoms with a structured diagnostic interview for mental disorders in children. Front Psychol. 2017;8 Available from: http://journal.frontiersin.org/article/10.3389/fpsyg.2017.00404/full. Cited 2021 Oct 14. 10.3389/fpsyg.2017.00404 PMC5366335 28396644

[61] Neuschwander M, In-Albon T, Adornetto C, Roth B, Schneider S (2013). Interrater-Reliabilität des Diagnostischen Interviews bei psychischen Störungen im Kindes- und Jugendalter (Kinder-DIPS). Z Kinder Jugendpsychiatr Psychother.

[62] Adornetto C, In-Albon T, Schneider S (2008). Diagnostik im Kindes- und Jugendalter anhand strukturierter Interviews: Anwendung und Durchführung des Kinder-DIPS. Klin Diagn Eval.

[63] First MB, Williams JBW, Karg RS, Spitzer RL (2016). SCID-5-CV: structured clinical interview for DSM-5 disorders: clinician version.

[64] Beesdo-Baum K, Zaudig M, Wittchen H-U, First v MB, Williams JBW, Benjaming LS, Spitzer RL (2019). Strukturiertes Klinisches Interview für DSM-5-Persönlichkeitsstörungen (SCID-5-PD). Deutsche Bearbeitung des Structured Clinical Interview for DSM-5-Personality Disorders.

[65] Cavelti M, Thompson K, Chanen AM, Kaess M (2021). Psychotic symptoms in borderline personality disorder: developmental aspects. Curr Opin Psychol.

[66] Chanen AM, Jovev M, Djaja D, McDougall E, Yuen HP, Rawlings D (2008). Screening for borderline personality disorder in outpatient youth. J Pers Disord.

[67] Fonagy P, Speranza M, Luyten P, Kaess M, Hessels C, Bohus M (2015). ESCAP Expert Article: borderline personality disorder in adolescence: an expert research review with implications for clinical practice. Eur Child Adolesc Psychiatry.

[68] Cloitre M, Shevlin M, Brewin CR, Bisson JI, Roberts NP, Maercker A (2018). The International Trauma Questionnaire: development of a self-report measure of ICD-11 PTSD and complex PTSD. Acta Psychiatr Scand.

[69] Haselgruber A, Sölva K, Lueger-Schuster B (2020). Symptom structure of ICD-11 Complex Posttraumatic Stress Disorder (CPTSD) in trauma-exposed foster children: examining the International Trauma Questionnaire – Child and Adolescent Version (ITQ-CA). Eur J Psychotraumatol.

[70] Brand RM, Bendall S, Hardy A, Rossell SL, Meyer D, Thomas N (2020). Moment-to-moment associations between posttraumatic stress symptoms and auditory hallucinations in the flow of daily life. Psychiatry Res.

[71] Schutte MJL, Linszen MMJ, Marschall TM, Ffytche DH, Koops S, van Dellen E (2020). Hallucinations and other psychotic experiences across diagnoses: a comparison of phenomenological features. Psychiatry Res.

[72] Sachser C, Berliner L, Holt T, Jensen TK, Jungbluth N, Risch E (2017). International development and psychometric properties of the Child and Adolescent Trauma Screen (CATS). J Affect Disord.

[73] Bernstein DP, Fink L, Handelsman L, Foote J, Lovejoy M, Wenzel K (1994). Initial reliability and validity of a new retrospective measure of child abuse and neglect. Am J Psychiatry.

[74] Wingenfeld K, Spitzer C, Mensebach C, Grabe H, Hill A, Gast U (2010). Die deutsche Version des Childhood Trauma Questionnaire (CTQ): Erste Befunde zu den psychometrischen Kennwerten. Psychother Psych Med.

[75] Stiglmayr C, Schimke P, Wagner T, Braakmann D, Schweiger U, Sipos V (2010). Development and psychometric characteristics of the dissociation tension scale. J Pers Assess.

[76] Woodward TS, Jung K, Hwang H, Yin J, Taylor L, Menon M (2014). Symptom dimensions of the psychotic symptom rating scales in psychosis: a multisite study. Schizophr Bull.

[77] Kronmüller K-T, von Bock A, Grupe S, Büche L, Gentner NC, Rückl S (2011). Psychometric evaluation of the Psychotic Symptom Rating Scales. Compr Psychiatry.

[78] Chandwick P, Lees S, Birchwood M (2000). The revised Beliefs About Voices Questionnaire (BAVQ-R). Br J Psychiatry.

[79] Strauss C, Hugdahl K, Waters F, Hayward M, Bless JJ, Falkenberg LE (2018). The Beliefs about Voices Questionnaire – revised: a factor structure from 450 participants. Psychiatry Res.

[80] Hayward M, Denney J, Vaughan S, Fowler D (2008). The voice and you: development and psychometric evaluation of a measure of relationships with voices. Clin Psychol Psychother.

[81] Lovibond SH, Lovibond PF (1995). Manual for the depression anxiety stress scales.

[82] Antony MM, Bieling PJ, Cox BJ, Enns MW, Swinson RP (1998). Psychometric properties of the 42-item and 21-item versions of the Depression Anxiety Stress Scales in clinical groups and a community sample. Psychol Assess.

[83] Henry JD, Crawford JR (2005). The short-form version of the Depression Anxiety Stress Scales (DASS-21): construct validity and normative data in a large non-clinical sample. Br J Clin Psychol.

[84] Ng F, Trauer T, Dodd S, Callaly T, Campbell S, Berk M (2007). The validity of the 21-item version of the Depression Anxiety Stress Scales as a routine clinical outcome measure. Acta Neuropsychiatr.

[85] R Core Team (2020). R: a langugae and environment for statistical computing.

[86] Spanakis G, Weiss G, Boh B, Roefs A, Zheng X, Zeng DD, Chen H, Leischow SJ (2016). Network analysis of ecological momentary assessment data for monitoring and understanding eating behavior. Smart Health.

[87] Wilkinson MD, Dumontier M, Aalbersberg IJ, Appleton G, Axton M, Baak A (2016). The FAIR Guiding Principles for scientific data management and stewardship. Sci Data.

