# Supplementary material for: Smartphone-assisted guided self-help cognitive behavioral therapy for young people with distressing voices (SmartVoices): study protocol for a randomized controlled trial
Source: Trials. 2022 Oct 23;23:902. doi: 10.1186/s13063-022-06846-0 (PMC9590132; doi:10.1186/s13063-022-06846-0)
Supplement: Supplementary file 1 — Additional file 1. Smartphone-based EMA items. Smartphone-based EMI items. [file 13063_2022_6846_MOESM1_ESM.docx]

**Additional file 1**

**Smartphone-based EMA items**

| **No.** | **Item** | **Response** | **Branching** | |
| --- | --- | --- | --- | --- |
| **Section A** | | | | |
| 1 | How did you sleep last night? *(presented only at the first assessment of the day)* | 1 (very bad) – 7 (very well) |  | |
| 2 | Right now I’m feeling … | 1 (satisfied) – 7 (dissatisfied) |  | |
| 3 | Right now I’m feeling … | 1 (uneasy) – 7 (at ease) |  | |
| 4 | Right now I’m feeling … | 1 (relaxed) – 7 (tense) |  | |
| 5 | Right now I’m feeling … | 1 (restless) – 7 (relaxed) |  | |
| **Section B** | | | | |
| 1 | Right now I’m happy with myself. | 1 (not true at all) – 7 (completely true) |  | |
| 2 | Right now I don't think anything good about myself. | 1 (not true at all) – 7 (completely true) |  | |
| **Section C** | | | | |
| 1 | Since the last beep, I have heard a voice or voices that others do not hear. | 1 (not true at all) – 7 (completely true) | If 1, continue with Section D, then with Section E  If ≥ 2, continue with Section C, then with Section E | |
| *Instruction:* If you have heard more than one voice since the last beep, please answer the following questions in relation to the voice that was the most difficult for you. | | | | |
| 2 | Right now, I feel bothered by the voice. | 1 (not true at all) – 7 (completely true) |  | |
| 3 | Right now, I have the feeling my voice wanted to harm me. | 1 (not true at all) – 7 (completely true) |  | |
| 4 | Right now, I have the feeling that my voice rules my life. | 1 (not true at all) – 7 (completely true) |  | |
| 5 | Right now, I have the feeling that I have no control over my voice. | 1 (not true at all) – 7 (completely true) |  | |
| 6 | Right now, I feel inferior to my voice. | 1 (not true at all) – 7 (completely true) |  | |
| 7 | Since the last beep, I have told my voice to shut up. | 1 (not true at all) – 7 (completely true) |  | |
| 8 | Since the last beep, I have yelled at my voice. | 1 (not true at all) – 7 (completely true) |  | |
| 9 | Since the last beep, I have done what my voice wanted me to do, even though I disagreed. | 1 (not true at all) – 7 (completely true) |  | |
| 10 | Since the last beep, I have told myself my voice is right, although I actually don't believe so. | 1 (not true at all) – 7 (completely true) |  | |
| 11 | Since the last beep, I have stepped up for myself when my voice treated me badly. | 1 (not true at all) – 7 (completely true) |  | |
| 12 | Since the last beep, I have defended my view against my voice. | 1 (not true at all) – 7 (completely true) |  | |
| **Section D** | | | |  |
| 1 | Right now, I am relieved not to hear any voices. | 1 (not true at all) – 7 (completely true) |  | |
| 2 | Right now, I feel lonely without my voices. | 1 (not true at all) – 7 (completely true) |  | |
| 3 | Right now, I feel lost without my voices. | 1 (not true at all) – 7 (completely true) |  | |
| 4 | Right now, I'm worried that my voices might come back. | 1 (not true at all) – 7 (completely true) |  | |
| **Section E** | | | |  |
| 1 | Since the last beep, I have been in touch with other people. | □ No, I haven’t | Continue with Section F, then with Section G | |
|  |  | □ Yes, with one person | Continue with Section E, then with Section G | |
|  |  | □ Yes, with two or more persons | Continue with Section E, then with Section G  *Instruction:* Please answer the following questions in relation to the person who was the most difficult for you to deal with. | |
| 2 | Meeting this person was for me ... | 1 (unpleasant) – 7 (pleasant) |  | |
| 3 | Right now, I feel inferior to this person. | 1 (not true at all) – 7 (completely true) |  | |
| 4 | Since the last beep, I have told this person to shut up. | 1 (not true at all) – 7 (completely true) |  | |
| 5 | Since the last beep, I have yelled at this person. | 1 (not true at all) – 7 (completely true) |  | |
| 6 | Since the last beep, I have done what this person wanted me to do, even though I disagreed. | 1 (not true at all) – 7 (completely true) |  | |
| 7 | Since the last beep, I have told myself this person is right, although I actually don't believe so. | 1 (not true at all) – 7 (completely true) |  | |
| 8 | Since the last beep, I have stepped up for myself when this person treated me badly. | 1 (not true at all) – 7 (completely true) |  | |
| 9 | Since the last beep, I have defended my view against this person. | 1 (not true at all) – 7 (completely true) |  | |
| **Section F** | | | |  |
| 1 | Right now, I am relieved to be on my own. | 1 (not true at all) – 7 (completely true) |  | |
| 2 | Right now, I feel lonely. | 1 (not true at all) – 7 (completely true) |  | |
| 3 | Right now, I feel lost. | 1 (not true at all) – 7 (completely true) |  | |
| 4 | Right now, I am worried that others don't want to be around me. | 1 (not true at all) – 7 (completely true) |  | |
| **Section G** | | | |  |
| 1 | Since the last beep, I have had the feeling of not being real or not feeling myself. | 1 (not true at all) – 7 (completely true) |  | |
| 2 | Since the last beep, I have had to think about negative experiences in the past. | 1 (not true at all) – 7 (completely true) |  | |
| 3 | Since the last beep, I have deliberately hurt myself without wanting to die. | □ Yes  □ No |  | |
| 4 | Since the last beep, I have thought about killing myself. | 1 (not true at all) – 7 (completely true) | If ≥ 4, show emergency number:  For adolescents up to 17 years: +41 (0)31 930 99 96 (7 days / 24h).  For adults from 18 years: +41 (0)31 632 88 11 (7 days / 24h) | |
| 5 | Since the last beep, I have drunk alcohol. | □ No  □ Yes, 1-2 glasses  □ Yes, 3-4 glasses  □ Yes, ≥ 5 glasses |  | |
| 6 | Since the last beep, I have used drugs. | □ No  □ Yes, cannabis  □ Yes, other drugs |  | |

**Smartphone-based EMI items**

| **No.** | **Item** | **Response** | **Branching** |
| --- | --- | --- | --- |
| **Section A** | | |  |
| 1 | Since the last beep, I have done the exercise that was proposed to me. | □ Yes | Continue with item 2, then with Section B |
|  |  | □ No, I didn’t do the exercise | Continue with items 3-7, then with Section B |
|  |  | □ No, no exercise was proposed to me | Continue with Section B |
| 2 | How helpful did you find the exercise? | 1 (not helpful at all) – 7 (very helpful) |  |
| 3 | I did not do the exercise because I did not have time. | 1 (not true at all) – 7 (completely true) |  |
| 4 | I did not do the exercise because I felt too miserable. | 1 (not true at all) – 7 (completely true) |  |
| 5 | I did not do the exercise because I forgot. | 1 (not true at all) – 7 (completely true) |  |
| 6 | I did not do the exercise because I did not feel like it. | 1 (not true at all) – 7 (completely true) |  |
| 7 | I did not do the exercise because I did not believe it would help me. | 1 (not true at all) – 7 (completely true) |  |
| **Section B** | | |  |
| 1 | Right now I’m happy with myself. | 1 (not true at all) – 7 (completely true) |  |
| 2 | Right now I don't think anything good about myself. | 1 (not true at all) – 7 (completely true) |  |
| **Section C** | | |  |
| 1 | Since the last beep, I have heard a voice or voices that others do not hear. | 1 (not true at all) – 7 (completely true) | If 1, continue with Section D, then with Section E  If ≥ 2, continue with Section C, then with Section E |
| *Instruction:* If you have heard more than one voice since the last beep, please answer the following questions in relation to the voice that was the most difficult for you. | | | |
| 2 | Right now, I feel bothered by the voice. | 1 (not true at all) – 7 (completely true) |  |
| 3 | Right now, I have the feeling my voice wanted to harm me. | 1 (not true at all) – 7 (completely true) |  |
| 4 | Right now, I have the feeling that my voice ruled my life. | 1 (not true at all) – 7 (completely true) |  |
| 5 | Right now, I have the feeling that I had no control over my voice. | 1 (not true at all) – 7 (completely true) |  |
| 6 | Right now, I feel inferior to my voice. | 1 (not true at all) – 7 (completely true) |  |
| 7 | Since the last beep, I have told my voice to shut up. | 1 (not true at all) – 7 (completely true) |  |
| 8 | Since the last beep, I have yelled at my voice. | 1 (not true at all) – 7 (completely true) |  |
| 9 | Since the last beep, I have done what my voice wanted me to do, even though I disagreed. | 1 (not true at all) – 7 (completely true) |  |
| 10 | Since the last beep, I have told myself my voice is right, although I actually don't believe so. | 1 (not true at all) – 7 (completely true) |  |
| 11 | Since the last beep, I have stepped up for myself when my voice treated me badly. | 1 (not true at all) – 7 (completely true) |  |
| 12 | Since the last beep, I have defended my view against my voice. | 1 (not true at all) – 7 (completely true) |  |
| **Section D** | | |  |
| 1 | Since the last beep, I have been in touch with other people. | □ No, I haven’t | End of the survey |
|  |  | □ Yes, with one person | Continue with Section D |
|  |  | □ Yes, with two or more persons | Continue with Section D  *Instruction:* Please answer the following questions in relation to the person who was the most difficult for you to deal with. |
| 2 | Meeting this person was for me ... | 1 (unpleasant) – 7 (pleasant) |  |
| 3 | Right now, I feel inferior to this person. | 1 (not true at all) – 7 (completely true) |  |
| 4 | Since the last beep, I have told this person to shut up. | 1 (not true at all) – 7 (completely true) |  |
| 5 | Since the last beep, I have yelled at this person. | 1 (not true at all) – 7 (completely true) |  |
| 6 | Since the last beep, I have done what this person wanted me to do, even though I disagreed. | 1 (not true at all) – 7 (completely true) |  |
| 7 | Since the last beep, I have told myself this person is right, although I actually don't believe so. | 1 (not true at all) – 7 (completely true) |  |
| 8 | Since the last beep, I have stepped up for myself when this person treated me badly. | 1 (not true at all) – 7 (completely true) |  |
| 9 | Since the last beep, I have defended my view against this person. | 1 (not true at all) – 7 (completely true) |  |
